# Poison

**Published:** 1879-10

**Authors:** 


POISON. It has been before stated that a prominent citizen was advised to apply a bit of candle grease to a pimple on his child's shoulder, he did so, and the child died in convulsions the next day, most likely the result of some chemical change arising from the contact of hot tallow with a brass candlestick. Many are carried away with "simple" remedies, that is, remedies composed of things with which they are familiar, and which at first sight would seem to be inert. The remedies for cough, cold and consumption, are innumerable, the combinations of ingredients are infinite; but if the reader is observant, not one in a hundred will there be which has not opium in the form of paregoric, laudnum, or morphia, giving water on the brain every year to multitudes of children and apoplexies or ruinous results to the digestive organs of adults. The life of Washington Irving was cut short by the injudicious recommendation of a simple cough-mixture by some pestiferous busybody. In any company of a dozen persons if one complains of anything from the scratch of a pin to a cancer, enough remedies will be volunteered in five minutes to kill a regiment of common men, advised too, with all the confidence that it is possible for ignorance to possess, for these two characteristics always exist in identical proportions; the greater the ignorance, the greater the certainty. The man who insures a cure of any thing under all circumstances is an ignoramus or a knave.

